# Brain Endothelium: The “Innate Immunity Response Hypothesis” in Cerebral Malaria Pathogenesis

**DOI:** 10.3389/fimmu.2018.03100

**Published:** 2019-01-29

**Authors:** Teresa F. Pais, Carlos Penha-Gonçalves

**Affiliations:** Instituto Gulbenkian de Ciência, Oeiras, Portugal

**Keywords:** cerebral malaria, endothelial cells, innate immunity, PAMPs, PRRs, blood-brain barrier

## Abstract

Cerebral malaria (CM) is a life-threatening neurological syndrome caused by *Plasmodium falciparum* infection afflicting mainly children in Africa. Current pathogenesis models implicate parasite and host-derived factors in impairing brain vascular endothelium (BVE) integrity. Sequestration of *Plasmodium*-infected red blood cells (iRBCs) in brain microvessels is a hallmark of CM pathology. However, the precise mechanisms driving loss of blood-brain barrier (BBB) function with consequent brain injury are still unsettled and it is plausible that distinct pathophysiology trajectories are involved. Studies in humans and in the mouse model of CM indicate that inflammatory reactions intertwined with microcirculatory and coagulation disturbances induce alterations in vascular permeability and impair BBB integrity. Yet, the role of BVE as initiator of immune responses against parasite molecules and iRBCs is largely unexplored. Brain endothelial cells express pattern recognition receptors (PRR) and are privileged sensors of blood-borne infections. Here, we focus on the hypothesis that innate responses initiated by BVE and subsequent interactions with immune cells are critical to trigger local effector immune functions and induce BBB damage. Uncovering mechanisms of BVE involvement in sensing *Plasmodium* infection, recruiting of immune cells and directing immune effector functions could reveal pharmacological targets to promote BBB protection with potential applications in CM clinical management.

## Introduction

Malaria is caused by *Plasmodium* parasites and transmitted to humans by the bite of an infective *Anopheles* mosquito. In 2016 over 200 million people were affected by malaria worldwide with approximately half million fatal cases occurring mostly in sub-Saharan Africa (World Malaria Report 2017). Malaria manifestations cover a wide clinical spectrum ranging from asymptomatic infection to life-threatening syndromes. The majority of fatality cases results from cerebral malaria (CM) that mainly afflicts children under 5 years of age infected with *Plasmodium falciparum*. Clinically, CM presents as an encephalopathy characterized by unarousable coma in patients carrying blood asexual forms of the parasite (1). Within children who survive CM, 10–20% will have sustained neurological sequels that may affect cognition, behavior, vision, hearing, and smell (2).

CM develops in approximately 1% of children infected with *P. falciparum* in endemic areas of Africa (3), likely determined by host genetics and parasite virulence factors. The neurological symptoms appear during blood stage infection when the parasite invades and multiplies within RBCs. During maturation, intra-erythrocytic parasites export proteins that extensively modify the host cell membrane taking part in iRBCs adherence to endothelial cells in blood capillaries and post-capillary venules of the brain (4). Members of the *P. falciparum* erythrocyte membrane protein 1 (PfEMP1) family mediate adherence of iRBCs to endothelial cells by binding to receptors such as cluster of differentiation 36 (CD36) (5), intercellular adhesion molecule 1 (ICAM-1) (5) and the endothelial protein C receptor (EPCR) (6). Indeed, sequestration of iRBCs in the cerebral microvasculature has emerged as the primary pathophysiological event in CM from post-mortem histological studies (7–9). Less is known about the secondary disease mechanisms underlying endothelium dysfunction, brain vasculopathy, parenchymal damage, as well as neurological sequels in those who recover from CM. At the single patient level the balance between anti-parasite responses and tissue protection mechanisms is a strong determinant of infection clinical outcomes (10). In fact, different pathogenesis mechanisms have been proposed to explain CM development. According to the “**hemodynamic hypothesis**,” adherence of iRBCs to the BVE leads to blood flow obstruction in microvessels causing hypoxia, nutrient deprivation and metabolic disturbances in adjacent brain tissue. Loss of cellular energy disturbs the membrane potential increasing intracellular water levels, a possible cause of cytotoxic brain edema in CM (11, 12). The “**inflammation hypothesis**” proposes that increased circulating levels of pro-inflammatory cytokines (e.g., TNF, IL1β, and IFNγ) in *P. falciparum*-infected patients induce the expression of adhesion molecules in the brain endothelium—i.e., ICAM-1, vascular cell adhesion molecule 1 (VCAM-1), and E-selectin (13, 14). Combined effects of iRBCs sequestration and systemic inflammation further enhance adherence of iRBCs and leukocytes to the BVE, agglutination between iRBCs and non-iRBCs (rosetting) (15) and platelet-mediated clumping of iRBCs (12). This leads to excessive endothelial activation with local secretion of proinflammatory mediators (16) and imbalanced release of vasoactive mediators, including endothelin-1 and angiopoietin-2 (17–19). Ultimately, the brain endothelial cell barrier becomes impaired with vascular leakage to the brain parenchyma.

As a consequence of endothelial activation patients with CM often show increased serum levels of the pro-coagulant von Willebrand factor (vWF) (20, 21) and tissue factor (TF) (22) offering support for a “**coagulation dysfunction hypothesis**” in CM development (23, 24). vWF is normally stored in intracellular organelles known as Weibel-Palade (WP) bodies but once secreted it forms strings that recruit platelets to the surface of the endothelial cells enhancing iRBCs adherence and amplification of the coagulation cascade (23, 25, 26). In addition, *P. falciparum*-iRBCs may inhibit the activated protein C (APC) anti-coagulant and anti-inflammatory pathway by competing for EPCR ligation (6) further contributing to a pro-coagulation/pro-inflammation cycle that compromises brain endothelial cell function.

Clearly, CM pathophysiology involves a dialogue between iRBCs and BVE mediated by parasite components and host-derived factors. Nevertheless, how BVE sense and respond to *Plasmodium* components and integrate innate immune stimuli with other immune effector mechanisms has largely been neglected. In this review we propose an “**innate immunity response hypothesis**” that addresses the pathogenic role of innate immune responses initiated by the BVE.

### The Blood-Brain-Barrier and CM

The blood-brain-barrier (BBB) is a selective physical barrier integrated in the neurovascular unit, which couples vascular and neural functions (27). The BBB restricts the traffic of molecules between the blood and the brain interstitial fluid, playing a key role in maintaining brain homeostasis. Through specific membrane transporters it supplies the brain with essential nutrients while avoiding access of toxic compounds and promoting the efflux of many waste products of brain metabolism. BBB is formed by the brain endothelial cells surrounded by basement membrane, pericytes and the end-feet of perivascular astrocytes. The barrier properties rely on highly organized tight junctions sealing intercellular spaces between endothelial cells. These structures are formed by the transmembrane proteins occludin and claudins which connect with adaptor proteins in the cytoplasm including the zonula occludens protein 1 (ZO-1) (28). Both human and experimental CM studies have reported a decrease in endothelial tight-junction proteins (ZO-1 and occludin) (29, 30). Damage of the BBB allows leakage of plasma proteins and fluids into the perivascular and parenchymal extracellular spaces causing vasogenic edema, which in part explains brain swelling observed in CM (31). Moreover, release of parasite and inflammatory factors in the perivascular space allows activation of other brain cells such as pericytes, astrocytes and microglia. These cells may produce locally inflammatory and neurotoxic factors that change neuronal activity and may cause neurological impairment even in patients who recover from CM (14, 32).

### The Experimental Cerebral Malaria Model (ECM)

Post-mortem studies in human cerebral malaria (HCM) and analysis of the serum of CM patients have highlighted potential disease mechanisms that have been addressed in context of experimental models. The most extensively studied ECM model uses C57BL/6 mice infected with *P. berghei* ANKA iRBCs. Within 7–10 days post-infection, mice develop neurological signals resembling HCM such as ataxia, convulsion, paralysis and/or coma that culminates in death in 70–100% of mice. Mice that do not develop ECM die at a later stage with hyperparasitemia and anemia (33–35). Similarly to HCM, mice treated with anti-malarial drugs after onset of neurological symptoms recover from malaria but display long-lasting cognitive deficits (36). The presence of parasite in the mouse brain is critical to ECM development (37) and brain pathology correlates with BVE activation and iRBCs sequestration in brain capillaries (38). However, the level of microvessel congestion and iRBCs accumulation is lower than in human cerebral microvasculature of fatal CM cases. This may reflect differences in human and mouse microvessel anatomy as well as in erythrocyte cytoadherence conferred by *P. falciparum* vs. *P. berghei* infection.

Parasite-specific CD8^+^ T cells primed in the spleen at day 3–5 post infection and then recruited to the brain have a critical role in ECM brain pathology. CD8^+^ T cells recognizing parasite antigens cross-presented by endothelial cells display cytotoxic activity towards the BVE through the action of granzyme B/perforin systems (39). In some cases of HCM, lymphocytes and monocytes are also found in brain tissue (40, 41) but there is no evidence for a critical role of CD8^+^ T cells.

Nevertheless, BVE activation is a decisive pathogenesis event in HCM and ECM (2, 31, 32) leading to vascular dysfunction, brain swelling and consequent intracranial hypertension (31, 42, 43). Indeed, it has been proposed that respiratory arrest resulting from compression of the respiratory centers in the brainstem is one cause of death in children with CM (31) and in ECM (33). Importantly, alterations in the neurovascular unit drive significant axonal injury and demyelination both in HCM (1, 44) and ECM (38).

## Brain Vascular Endothelium (BVE): a Trigger in CM Immunopathology

### BVE as a Sensor of Circulating Plasmodium-Derived Factors

Endothelial cells are among the first cells exposed to systemic alterations caused by blood borne pathogens and the first defense against CNS infection. Similarly to cells of the innate immune system (e.g., macrophages, monocytes, neutrophils and dendritic cells), endothelial cells are equipped with pattern recognition receptors (PRRs) specialized in detecting pathogen-associated molecular patterns (PAMPs) and danger-associated molecular patterns (DAMPs). BVE expresses various Toll-like receptors (TLR1-10) (45) and NOD-like receptors (NLRs) (46) as well as retinoic acid inducible gene 1 (RIG-I) of the RIG-I-like receptors (RLRs) family (47) and the absent in melanoma 2 (AIM2) (46), a component of the inflammasome that recognize microbial nucleic acids (48).

BVE responds actively to microbial infection, which in some cases may compromise BBB and contribute to disease pathogenesis. As an example, TLR2 and TLR4 bind to bacterial components of meningococcal lysates inducing production of high levels of nitric oxide (NO), a highly reactive factor with antimicrobial activity but that is also cytotoxic for brain endothelial cells (49). It has also been shown that TLR2/6 signaling triggered by fungal zymosan induces tight junction disruption and increases permeability of the brain endothelial cell barrier *in vitro* (45). On the other hand, activation of TLR3 and RIG-1 in the BVE results in production of IFNs that inhibit HIV replication in macrophages (50). This illustrates that innate sensing by the BVE can have both beneficial and deleterious effects in brain pathology.

During *Plasmodium* infection, iRBCs and iRBCs-derived factors accumulate in the spleen and are sensed by innate immune recognition receptors strongly activating immune cells (51). But early during infection the BVE is also exposed to iRBC-derived factors and likewise responds to innate stimulation. In fact, exposing human brain microsvascular endothelial cells to *P. falciparum*-iRBCs leads to rapid nuclear translocation of p65 NF-kB subunit with transcriptional activation of NF-kB target genes (e.g., chemokines and pro-inflammatory cytokines) (52). Interestingly, this effect is independent of cytoadherence of *P. falciparum*-iRBCs to the endothelium suggesting a role for iRBCs-derived soluble factors in BVE activation (52). This is the case of the *P. falciparum* histidine-rich protein II (PfHRP-2), which is exported to the erythrocyte cytosol. Upon rupture of iRBCs, PfHRP-2 is released into the bloodstream as shown by increased plasma levels in CM patients (53). When added to brain endothelial cells PfHRP-2 induces IL-1β production and increases permeability of the endothelial barrier. These effects seem to depend on PfHRP-2 recognition by a receptor not yet identified that triggers the inflammasome. As a result, caspase-1 becomes activated and cleaves the pro-IL1β into its mature form (54).

Likewise, BVE could be activated by recognition of parasite-derived factors through PRRs known to operate in the innate immune cells (Table 1). Hemozoin —a product of host hemoglobin digestion by the parasite that accumulates in form of crystals— is recognized by TLR9 (55), and hemozoin crystals activate the NLRP3 inflammasome in macrophages and dendritic cells (57). Furthermore, plasmodial DNA bound to hemozoin may activate the TLR9 through recognition of CpG-containing motifs (56). Also, AT-rich plasmodial DNA regions may reach the cytosol via hemozoin and activate the stimulator of interferon genes (STING). This pathway leads to IFNβ induction through the activation of Tank binding kinase 1 (TBK1) and of interferon regulatory transcription factors IRF3 and IRF7 (58). It has been proposed that hemozoin bound to TLR9 may prime the cell for subsequent activation of the NLRP3 and AIM2 inflamasomes by recognition of hemozoin-bound plasmodial DNA and leading to release of pro-inflammatory IL1β (59). In dendritic cells and macrophages, *P. falciparum* glycosylphosphatidylinositol (Pf-GPI) anchors, which are structurally different from human GPI, are recognized by TLR2 heterodimers with TLR1 or TLR6 and activate transcription of inflammatory cytokines (TNF and IL1β) (60).

**Table 1:** Recognition of *Plasmodium* factors by innate immune cells.

***Plasmodium*-derived factors**	**Sensors**	**Cell types**	**References**
PfHRP-2	Unidentified	Brain microvascular cell line	(54)
Hemozoin (Hz) Hz/DNA	TLR9	Monocytes, macrophages, and dendritic cells	(55, 56)
	NLRP3		(57)
	STING		(58)
	AIM2		(59)
Pf-GPI	TLR2-TLR1 TLR2-TLR6	Monocytes and Macrophages	(60)
Microvesicles (MVs)	TLR4	Macrophages	(61)
Genomic DNA-MVs	cGAS-STING	Monocytes	(62)

Accumulating evidence suggests that increased plasma levels of microvesicles (MVs) released by different cell types during infection contribute to the pathogenesis of CM. MVs are heterogeneous populations of membranous extracellular vesicles (0.1–1 μm in size) that are formed by budding from the cell plasma membrane. They carry parasite-derived proteins, lipids, and nucleic acids that may be recognized by PRRs and other innate receptors possibly contributing to CM pathogenesis through the activation of inflammatory pathways (63). MVs isolated from the plasma of malaria infected mice or from *P. falciparum*-iRBCs cultures induce a pro-inflammatory response in macrophages (61, 64), namely through binding to TLR4 (61). Interestingly, iRBCs-derived extracellular vesicles contain parasite genomic DNA that binds to cyclic GMP-AMP synthase (cGAS) inducing type I IFN in human monocytes through the cGAS-STING pathway (62). These lines of evidence illustrate that parasite-derived factors such as hemozoin and MVs are inducers of innate immunity pathways namely by carrying into the cell plasmodial nucleic acids and allowing recognition by intracellular receptors. We propose that these receptors are potential candidates for parasite sensing and activation of the BVE subsequently leading to BBB disruption during CM.

### Brain Endothelial Cells as Recruiters and Capturers of Leukocytes

Under basal conditions endothelial cells express low mRNA levels of several cytokines and chemokines. However, different stimuli including hypoxia and exposure to microbial products trigger a pro-inflammatory response by endothelial cells with secretion of pro-inflammatory mediators that in turn fuel vascular inflammation (65). Chemokines are small cytokines able to mediate chemotaxis of immune cells by signaling through chemokine receptors (66). This is a pivotal mechanism to recruit lymphocytes and monocytes to sites where tissue is damaged by infection. *In vitro* cell culture systems have shown that *P. falciparum*-iRBCs increase the transcription of several chemokines in human brain endothelial cells (CXCL8, CXCL1, CXCL2, CCL20, and CCL2/MCP1) (52, 67). Interestingly, several human studies identified the C-X-C motif chemokine 10 (CXCL10) as a biomarker of CM and a predictor of mortality (68). These findings should encourage research on the role of endothelial chemokines in leukocyte recruitment in HCM. CXCL10 is a chemoattractant for monocytes, neutrophils, NKT cells and activated effector CD4^+^ and CD8^+^ T lymphocytes signaling through the cell surface C-X-C motif chemokine receptor 3 (CXCR3) (66). Expression of CXCL10 and CXCR3 is involved in ECM pathogenesis as shown by the increased protection in CXCL10 KO and CXCR3 KO mice (69). In competitive cell transfer, CXCR3-deficient CD8^+^ T cells were less efficiently recruited to the brain of *P. berghei* ANKA-infected mice than wild-type cells. CXCL10 gene transcription is highly induced in the brains of mice with CM (~200-fold) (69) but only recently, using reporter mice that drive the expression of fluorescent protein under the CXCL10 promoter it became clear that *P. berghei* ANKA infection strongly up-regulates CXCL10 in brain endothelial cells localized in post-capillary venules. Multiphoton intravital microscopy further evidenced that endothelial CXCL10 promotes adhesion of T cells to the brain vasculature in *P. berghei* ANKA-infected mice (70). These observations fully support the hypothesis that parasite sensing by the BVE is critical in mounting an adaptive effector immune response in the brain vasculature.

In addition to recruitment the BVE also sequesters CD8^+^ T cells in the brain through binding of ICAM-1 and VCAM-1 to their counter-ligands on recruited CD8^+^ T cells, the lymphocyte function-associated antigen (LFA-1) and very late activation antigen-4 (VLA-4), respectively. Blocking of this interaction at late stages of infection prevents CM in infected mice (30). Further, we have shown that IFN (α/β) receptor 1 (IFNAR1) expression on CD8^+^ T cells is required for cells to induce BBB disruption in CM (35) and it is plausible that IFNα/β produced in the brain endothelium triggers IFNAR1 signaling on CD8^+^ T cells licensing cytotoxic activity.

### Brain Endothelial Cells as Presenters of Plasmodium-Derived Antigens

Endothelial cells are not professional antigen-presenting cells (APCs) but under certain inflammatory conditions they present major histocompatibility complex (MHC) class I and class II antigens inducing proliferation of CD4^+^ and CD8^+^ memory T cells (65).

In experimental CM, CD8^+^ T cells proliferate in the brain (71) and parasite-specific CD8^+^ T cells (Granzyme B^+^/perforin^+^) recruited to the brain only become pathogenic if the parasite biomass reaches a certain threshold (37, 72). This suggests that cytotoxic CD8^+^ T cells only exert their effector functions above a given level of presentation of MHC class I-restricted parasite peptides in the brain. Combined *in silico* analysis of *P. berghei* proteome data (73) and TCR sequences of brain-sequestered CD8^+^ T cells (39) allowed prediction of immunogenic class I-restricted parasite peptides. Nevertheless, MHC I tetramers bound to these different parasite peptides only identified the specificity of approximately 12% of the CD8^+^ T activated cells in the brain suggesting that CD8^+^ T cells with yet unknown antigen specificities are involved in CM (72, 74).

Evidence of cross-presentation of parasite antigens by endothelial cells was provided using a NFAT-LacZ reporter cell line transgenic for a TCR that recognizes a *P. berghei* peptide in the context of MHC class I. This cell line allowed detection of antigen presentation by endothelial cells isolated from *P. berghei*-infected mouse brains and by cultures of murine brain endothelial cells stimulated with IFNγ and exposed to iRBCs or free merozoites. Primary endothelial cells were unable to present merozoite-derived antigens and activate reporter cells in the absence of the transporter associated with antigen processing (TAP)—transporter of MHC class I-peptide loaded complexes—or under proteasome inhibition, indicating that brain endothelial cells acquire and process malaria antigens that are subsequently cross-presented to CD8^+^ T cells (60).

It has been suggested that merozoites and digestive vacuoles rather than whole iRBCs are the source of parasite antigens processed by endothelial cells (75). Another study showed that iRBC membrane components are transferred to the membrane of endothelial cells (trogocytosis-like mechanism) followed by engulfment of the iRBCs by a cup-like structure. This mechanism allows recycling of parasite material to the endosomal and cytosolic compartments of endothelial cells (76). Interestingly, this process of antigen uptake leads to disruption of endothelial tight-junctions and decrease in transendothelial electric resistance in absence of lymphocytes, suggesting that uptake of parasite components exerts direct detrimental effects in the endothelial barrier that are possibly operating in CM development (76).

Finally, *in vivo* imaging corroborates that *Plasmodium*-coded antigens are cross-presented by brain endothelial cells. In mice infected with *P. berghei*-OVA observation of ovalbumin-specific CD8^+^ T cells (OT-I T cells) arresting and interaction with the BVE was dependent on MHC class I-restricted antigen presentation (30). Nevertheless, the identity of parasite antigens cross-presented by the brain endothelial cells remains largely unknown. The analysis of the mechanisms underlying the acquisition of *Plasmodium* antigens by the BVE could provide useful information on the range of antigens involved in CM development and would benefit preventive interventions directed to deter immunological-mediated BVE damage.

## Concluding Remarks

**Figure 1:**
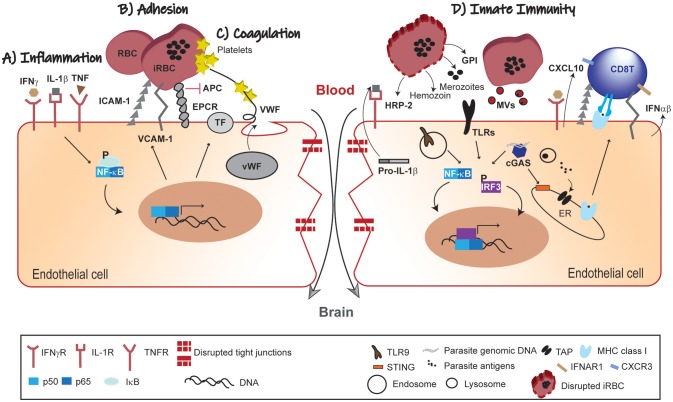
Pathophysiology of BBB leakage in CM. **(A)** Inflammation; the systemic immune response against the malaria parasite increases circulating levels of pro-inflammatory cytokines. In response to this inflammatory mellieu NF-κB-dependent gene transcription of adhesion molecules and pro-coagulation factors is activated in BVE. **(B)** Adhesion; iRBCs adherence to BVE via adhesion molecules (ICAM-1 and VCAM-1) compromises microvascular blood-flow. **(C)** Coagulation; secretion of pro-coagulation factors (TF and vWF), platelets aggregation and competitive inhibition of EPCR by iRBCs converge in promoting intravascular coagulation. These mechanisms are interlinked such that endothelial activation by pro-inflammatory factors feeds-forward iRBCs adherence and contributes to coagulation dysfunction. **(D)** Innate Immunity; parasite-derived factors (HRP-2, GPI hemozoin and merozoites) recognized by PRRs activate both NF-κB and IRF3 transcription factors with production of chemokines (e.g., CXCL10), IFNα/β and increased expression of adhesion molecules. These signals contribute to the recruitment of leukocytes and take part in the cross-talk with the adaptive immune system, namely effector CD8^+^ T cells. Individually or in different combinations, these pathophysiological trajectories lead to disruption of the endothelial barrier and dysfunction of the BBB in CM. IFNγ, interferon gamma; IL, interleukin; TNF, tumor necrosis factor; IFNγR, IFNγ receptor; IL-1R, IL-1 receptor; TNFR, TNF receptor; ICAM-1, intercellular adhesion molecule-1; VCAM-1, vascular cell adhesion molecule 1; EPCR, endothelial protein C receptor; APC, activated protein C; TF, tissue factor; vWF, von Willebrand factor; NF-kB, nuclear factor kB; IkB, kB inhibitor; RBC, red blood cell; iRBC, infected-red blood cell; HRP-2, histidine-rich protein II; GPI, glycosylphosphatidylinositol; TLRs, Toll-like receptors; MVs, microvesicles; cGAS, cyclic GMP-AMP synthase; CXCL10, C-X-C motif chemokine 10; IFNα/β, interferon α/β; ER, endoplasmic reticulum; IRF3, interferon regulatory factor 3; STING, stimulator of interferon genes; TLR9; Toll-like receptor 9; TAP, transporter associated with antigen processing; IFNAR1, IFN (α/β) receptor 1; MHC class I, major histocompatibility complex class I; CXCR3, C-X-C motif chemokine receptor 3.

Here, we highlighted the role of BVE activation at the crossroads of different CM pathophysiological trajectories that promote microvessel obstruction, intravascular coagulation and vascular leakage (Figures 1A–C). These vascular disturbances can contribute to cytotoxic or vasogenic edema, critical clinical events in CM severity. This supports the perspective that CM pathogenesis is heterogeneous comprising different pathogenic mechanisms that may concur to disease in the same individual or play distinct roles in different individuals.

In addition, we propose that the BVE is not only a target of ongoing inflammation, but actively senses parasite components using innate immunity pathways to recruit and deter immune cells in vascular sites of parasite accumulation. Moreover, BVE may trigger effector functions of the adaptive immune system as exemplified by the cytotoxic activity by CD8^+^ T cells in ECM (Figure 1D). Addressing this hypothesis will unravel the endothelial innate receptors and pathways involved in parasite sensing and in the crosstalk with adaptive immune cells. We anticipate that this research will provide additional diagnostic tools and pharmacological approaches to prevent BVE dysfunction and neurologic sequels of CM (77).

## Author Contributions

All authors listed have made a substantial, direct and intellectual contribution to the work, and approved it for publication.

### Conflict of Interest Statement

The authors declare that the research was conducted in the absence of any commercial or financial relationships that could be construed as a potential conflict of interest.
